# High-Precision and Low-Complexity Silicon Waveguide-Integrated Temperature Sensor System

**DOI:** 10.3390/s25133922

**Published:** 2025-06-24

**Authors:** Zhiming Zhang, Haole Kong, Yi Li

**Affiliations:** College of Optical and Electronic Technology, China Jiliang University, Hangzhou 310018, China; s22040803048@cjlu.edu.cn (Z.Z.); s22040803012@cjlu.edu.cn (H.K.)

**Keywords:** silicon waveguide, temperature sensor, convolutional neural network

## Abstract

This paper proposes a high-sensitivity-integrated temperature sensor with low complexity based on a silicon waveguide. The waveguide layout is optimized through the finite-difference time-domain (FDTD) simulations, and a compressed taper structure improves the efficiency of speckle data collection while reducing the system complexity and cost. To achieve precise temperature demodulation, this paper employed a convolutional neural network (CNN) for nonlinear fitting. Experimental results demonstrate the sensor’s ability to perform temperature measurement in the range of −20 °C to 100 °C, with a best resolution of 0.00287 °C (2.87 mK). The resolution and reliability of the measurements are validated by comparison with the theoretical values. This study introduces a novel approach to silicon waveguide-based temperature sensing.

## 1. Introduction

With the rapid advancement of science and technology, accurate temperature measurement has become crucial across various disciplines, in particular, the fields of aerospace, geophysics, industrial production, and biomedicine [1,2,3]. These applications demand sensors with high resolution, broad measurement range, high temperature resistance, and robust anti-interference capabilities. However, conventional electronic temperature sensors in special environments such as deep sea or aerospace often fail to meet the demanding requirements of such applications due to space constraints, lack of heat resistance, and susceptibility to electromagnetic interference.

In contrast, temperature sensors based on optical technology have emerged as promising solutions due to their superior resolution, electromagnetic immunity, and capacity for remote transmission [4,5,6]. Among these, Fiber Bragg Grating (FBG) sensors [7,8,9] and multimode fiber sensors [10,11] have been widely utilized in several application scenarios. However, these technologies still encounter certain limitations. For instance, the wavelength response bandwidth and the linear response range of FBG sensors impose inherent constraints. Moreover, the need for additional external instruments, such as swept light sources or spectrometers, increases the complexity and cost of these systems. Chen et al. [9] developed a π-phase-shifted fiber-grating temperature-sensing system, which requires a narrow linewidth swept light source, a matching hydrogen cyanide (HCN) gas cell, and a Mach–Zehnder interferometer (MZI) for high-precision temperature measurement. In contrast, multimode optical fiber sensors also face challenges in achieving the high measurement precision required for extreme conditions. These challenges often stem from environmental sensitivities and limited reliability over long distances. Consequently, there is a clear need for in-depth research into the design of a high-sensitivity, wide-range, and stable optical temperature sensor.

Recent advancements in fiber speckle sensing (FSS) offer a promising alternative to optical fiber sensing [12,13]. FSS leverages the intermodal interference of coherent light propagating through multimode fibers to produce speckle patterns at the fiber’s output. While traditionally considered modal noise in optical communication systems, these speckle patterns contain valuable information about the environment where the fiber is located. The sensing system is relatively simple, requiring only a coherent light source, a multimode fiber, and photodetector array or CCD. Despite its simplicity, FSS faces significant challenges, including high cross-sensitivity to various environmental factors such as physical vibrations, stress, temperature, refractive index changes, and biochemical interactions [14,15,16,17,18,19]. These sensitivities complicate the extraction of target physical quantities from unintended perturbations. Li et al. [20] used multiple probes to simultaneously measure temperature and refractive index, effectively distinguishing between the two. Nguyen et al. [21] came up with a novel idea of feeding broadband light into multimode optical fibers and applying random noise to the fibers, thus obtaining a large amount of speckle data, using dense neural network (DNN) training data to distinguish the noise. While effective, these approaches often involve considerable system complexity due to their reliance on wavelength domain techniques.

In recent years, silicon photonics has gained significant attention in optical sensing applications due to its excellent optical performance, integration capabilities, and stability. Current silicon-based optical temperature sensors often utilize micro-ring resonator structures where temperature-induced changes in resonance states enable measurement [22,23,24,25]. Wang et al. [25] achieved a temperature measurement range of −20 °C to 105 °C with a resolution of 60 mK using cascaded micro-ring arrays. Irace et al. [26] used bare silicon as a sensor device yet did not deviate from the basic structure of singlemode-multimode-singlemode (SMS), resulting in a relatively low measurement resolution.

This study explores a novel approach to silicon photonics-based temperature sensing using long multimode silicon waveguides. The proposed sensor leverages the thermo-optic effect of silicon, wherein temperature changes induce small but cumulative variations in the effective refractive index of propagation modes. When the multimode waveguide is sufficiently long, these variations significantly affect the speckle pattern, enabling highly sensitive temperature measurements. This work also employs a taper structure to compress speckle patterns into four-channel intensities, which are then upsampled and analyzed using convolutional neural networks (CNNs) for temperature demodulation. The experimental results show that the silicon chip with an area of less than 1 mm × 1 mm is able to achieve temperature measurement range from −20 °C to 100 °C with a best resolution of about 2.87 × 10^−3^ °C.

Our proposed silicon waveguide is a multimode waveguide, which is fundamentally different from conventional singlemode silicon waveguide. It utilizes fiber speckle sensing principles to achieve significantly higher temperature sensitivity compared to singlemode silicon devices. For instance, while the sensor reported in reference [25] demonstrates a temperature resolution of 60 mK, our proposed sensor achieves a resolution of 2.87 mK. Furthermore, unlike traditional micro-ring resonators or MZIs that require complex and expensive spectral acquisition systems (such as scanning lasers or spectrometers), our design employs just a single-wavelength semiconductor distributed feedback (DFB) laser and four photodetectors (PDs) for power measurement. It reduces both system complexity and financial cost.

## 2. Device and System Design

### 2.1. FDTD Simulation

**Figure 1:**
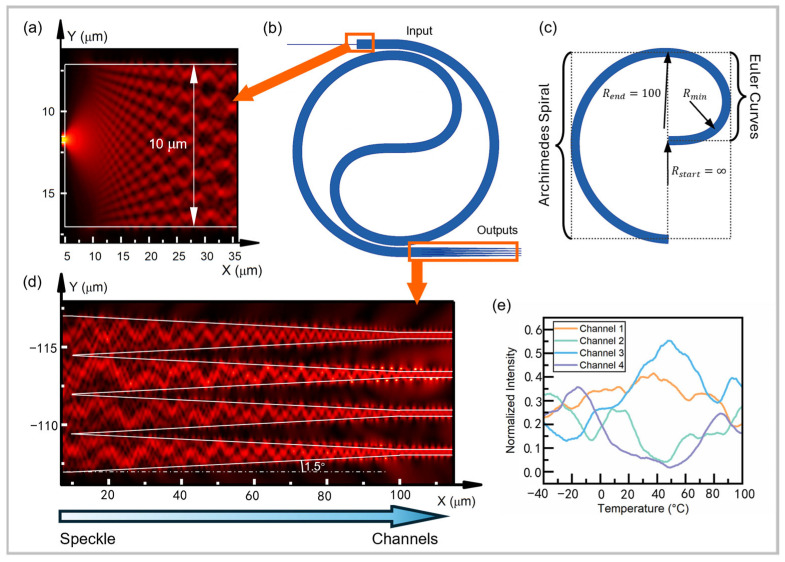
(**a**) Light intensity distribution of mode excitation at the connection of input singlemode and scattering multimode. (**b**) Schematic diagram of the smallest structure including singlemode waveguide, multimode waveguide, and taper output part. (**c**) Schematic diagram of the two Euler curves. (**d**) Light intensity distribution of the taper structure compressed the speckle pattern. (**e**) Simulation results of the four-channel light intensity obtained by the MONITOR showing pseudo-random but stable variation with the change in ambient temperature.

To facilitate the design and iteration, this study utilizes a commercial silicon photonics Multi Project Wafer (MPW) process. In this process, the top silicon waveguide features a thickness of 220 nm and a standard singlemode width of 450 nm, encapsulated within a silica cladding. To illustrate, we designed a minimum effective structure including input singlemode waveguide, scattering multimode waveguide and tapered output structure as shown in Figure 1a, and calculated the whole process of speckle formation and compression using FDTD simulation. We used the FDTD module of the Lumerical simulation software (version: Lumerical 2020 R2.3)developed by Ansys.

The input waveguide, with a width of w_0_ = 0.45 μm, directly connects to the multimode waveguide, which has a width of w_1_ = 10 μm. This arrangement maximizes the excitation of transmission modes and enhances the speckle formation process. Figure 1a illustrates the simulated light intensity distribution at the interface between the singlemode and multimode waveguides, confirming effective mode excitation.

In the coiled center section of the waveguide, two Euler curves are employed to connect the winding-in and winding-out portions of the waveguide (see Figure 1b). The radius of curvature decreases from R_start_ = ∞ in the center to a minimum value of R_min_ = 37.91, and then increases to R_end_ = 100 at the outer side. This property, which ensures that the curvature of the waveguide is continuous throughout, reduces the propagation loss and modal distortion in the central bending area [27].

In order to compress the speckle data more efficiently, the speckle is compressed into four-channel light intensity data using four taper structures with an angle of 1.5°, as shown in Figure 1d. This compression is crucial for reducing system complexity and enabling simplified data acquisition. Simulation results, as shown in Figure 1e, reveal that the compressed speckle intensity exhibits pseudo-random and nonlinear variations with ambient temperature changes. As the temperature changes, the refractive index and length of the waveguide will change, resulting in a change in the optical path. The change in the optical path leads to a change in the mode interference; thus, the speckle pattern also changes. The temperature can be demodulated by analyzing the speckle data.

Due to computational resource constraints, simulations were conducted on a waveguide with a length of 1 mm. Even at this reduced length, the speckle intensity demonstrates strong temperature-dependent characteristics. It is reasonable to assume that a longer waveguide would produce more pronounced speckle randomness, further enhancing temperature sensitivity.

### 2.2. Design and Packaging of Silicon Chip

**Figure 2:**
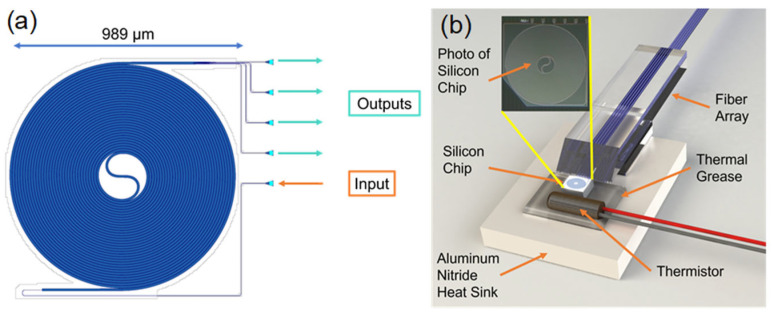
(**a**) Layout mask of the multimode waveguide. (**b**) Schematic diagram of the coupling scheme for the temperature sensing probes.

Multimode silicon waveguides differ from the actual simulation due to unavoidable process errors. Through the simulation, we obtained an acceptable range of waveguide lengths. We designed several chips with different lengths (the length range is approximately 20 mm to 60 mm) on the wafer. Finally, after testing, we selected the longest chip to be used in experiments. The chosen layout mask is shown in Figure 2a, configured as an Archimedean spiral with 34 coils, resulting in an approximate waveguide length of 62 mm. The input and output of the light are coupled by vertical grating couplers positioned on the same side of the chip. This configuration simplifies the coupling of the fiber arrays and improves the coupling efficiency.

Considering the actual application environment, temperature-resistant materials were selected for the coupling components, including fiber array (FA), fixing adhesive, and coupling adhesive, all capable of withstanding temperatures from −40 °C to 150 °C. While the chip’s compact size inherently supports miniaturization, an aluminum nitride heat sink was incorporated to ensure uniform temperature distribution across the device during experiments.

Given the nonlinear relationship between temperature and speckle pattern, preliminary calibration is essential. A thermistor is placed in close contact with the SOI chip, with the gap filled using thermally conductive adhesive to maintain consistent thermal conditions. This setup ensures that the temperature measured by the thermistor closely approximates the true temperature of the chip. An external temperature acquisition circuit records the thermistor’s temperature. Figure 2b shows the coupling and packaging scheme where the entire module functions as a sensing probe.

## 3. Experimental Results and Analysis

### 3.1. Measurement System Setup

**Figure 3:**
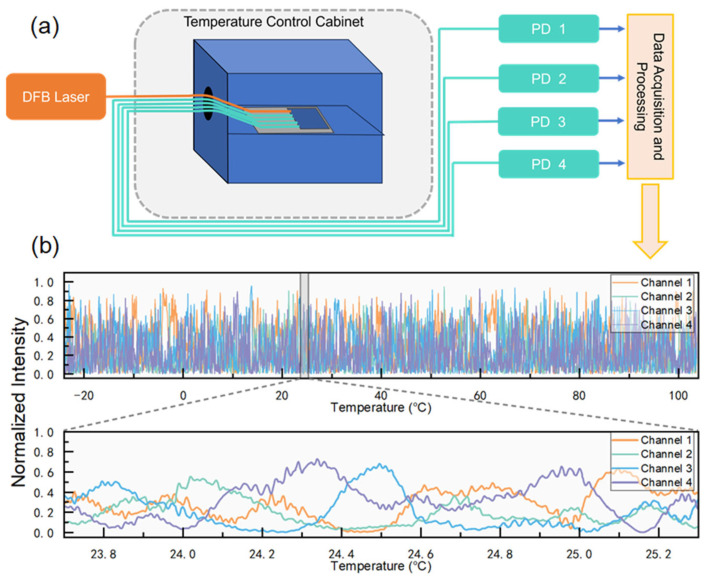
(**a**) Schematic diagram of the measurement system, showing the process of laser input, speckle formation, light intensity acquisition, and data uploading; (**b**) four-channel light intensity variation curve with temperature.

The measurement system designed in this study is notably simple, and its structure is illustrated in Figure 3a. A coherent light source is input into the sensing probe where a speckle is generated in the SOI. The speckle is subsequently compressed into four-channel light intensity data via a taper structure and then coupled back to the FA. The FA pigtail is connected to a photodetector array, which captures the signals and transmits them to a detection circuit, which has an ADC quantization accuracy of 16 bits. Meanwhile, the temperature of the thermistor is collected and calculated by the temperature measurement circuit. The resistance of the thermistor at 25 °C is 10 KΩ with a B value of 3950 K. B = [ln(R₁/R₂)]/[(1/T₁) − (1/T₂)]. R_1_ is the resistance of the thermistor at temperature T_1_ and R_2_ is the resistance at temperature T_2_. T_1_ and T_2_ are 25 °C and 85 °C (i.e., 298.15 K and 358.15 K). The ADC model is ADS9810 designed by Texas Instruments (manufacturer: Texas Instruments Semiconductor Manufacturing (Chengdu) Co., Ltd., Chengdu, China) with an accuracy of 18 bits and a sampling rate of 1 MHz. The temperature resolution of the temperature measurement circuit in the range of −20 °C to 100 °C is 0.001 °C, which was obtained by calculation. Finally, the data is uploaded to a computer for subsequent analysis and processing.

Unlike conventional systems that operate in the spectral domain and require complex components such as tunable lasers, this setup operates solely in the intensity domain. As a result, it only requires a DFB laser with an output wavelength of 1550 nm as the coherent light source.

### 3.2. Neural Network Algorithm for Demodulation

In the experiment, data collection for pre-calibration was a critical step. The encapsulated sensor probe was subjected to a series of temperature cycles inside a temperature control cabinet. Figure 3b displays the four-channel light intensity as a function of temperature across the range of −20 °C to 100 °C. The lower half of the figure provides a detailed view of the variation near room temperature.

The speckle intensity exhibits a markedly pronounced random variation with temperature. It is noteworthy that the “small waves” observed in the intensity curves are not noise but represent the intrinsic temperature-sensitive speckle variations. These variations, characterized by a steep average slope, indicate the high temperature sensitivity of the sensor.

To extract temperature data from the recorded data, a suitable demodulation algorithm was essential. Observing the significant nonlinearity in the mapping between temperature and the four-channel light intensity, a convolutional neural network (CNN) was selected for its superior capability in handling nonlinear relationships and processing high-dimensional data.

The preprocessing of the data involved upsampling to enhance the input dimensionality for the CNN. A 1 × 4 data input was upsampled to 32 × 15, following these steps:

1. Power features expansion: $f_{POW}=\cup_{i=3}^{77}x_{n}^{10i}$.
2. Exponential features expansion: $f_{exp}=\cup_{i=4}^{7}\frac{\exp\left(x^{0.1i}\right)}{\max\left(\exp\left(x^{0.1i}\right)\right)}$.
3. Interactive features: $f_{inter}$, including two-variable linear combinations, three-variable linear combinations, two-variable products, three-variable products, and interaction higher order features.
4. Feature integration: $f_{final}=x_{n}\cup f_{POW}\cup f_{exp}\cup f_{inter}$.
5. Data reconstruction: $F_{upsampled}=\mathrm{reshape}\left(f_{final},\left(35,12\right)\right).$

**Figure 4:**
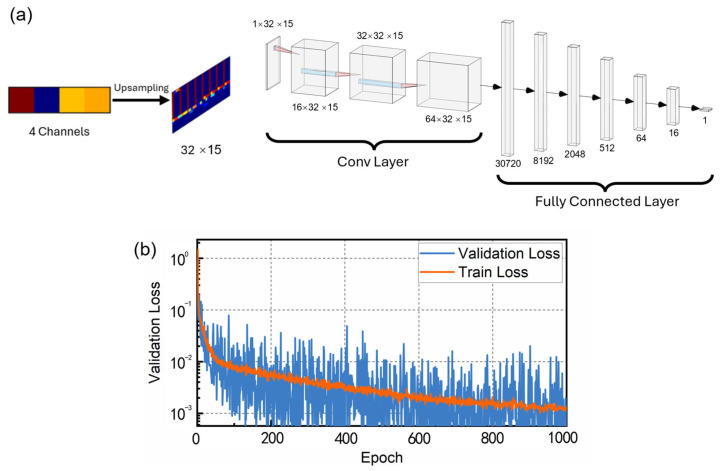
(**a**) CNN architecture used in the experiment; (**b**) training and validation RMSE curves during the training process.

The CNN architecture, as shown in Figure 4a, comprises three convolutional layers and five fully connected layers, with ReLU activation functions used throughout. The final output is a single node representing the temperature. The network was implemented using the PyTorch (version: 1.13.1) framework and trained on a NVIDIA GeForce RTX 3080 GPU.

The training data was generated from five temperature cycles spanning the range of −20 °C to 100 °C, resulting in 2.5 million data samples. These samples were upsampled and randomly shuffled. The root mean square error (RMSE) was used as the loss function, and the Adam optimizer was employed for model training. The specific parameters of the CNN architecture are as follows. The model is trained for up to 1000 epochs using an initial learning rate of 0.1, which is reduced by a factor of 0.1 whenever the validation loss fails to show significant improvement over 100 consecutive epochs. The training employs a batch size of 64 and incorporates L1 regularization to prevent overfitting. Early stopping is triggered if the loss fails to converge further after five rounds of learning rate decay. The dataset is partitioned into training (80%), validation (10%), and test (10%) subsets to ensure robust evaluation.

Figure 4b shows the evolution of the RMSE for both training and validation sets throughout 1000 epochs. The RMSE for the training set reached 10^−2^ °C after 50 iterations and converged to 0.00132 °C after 1000 iterations. The validation results indicate that the CNN model was effectively trained and exhibits strong generalization performance.

### 3.3. Measurement Resolution

#### 3.3.1. Temperature Control Cabinet Experiment

The temperature resolution of the temperature measurement system is first evaluated through experiments on demodulating the temperature of the temperature control cabinet (as shown in Figure 3a). Temperature measured by the thermistor is used as the actual temperature, and the result of the neural network demodulation algorithm is used as the demodulated temperature. Comparing the actual temperature with the demodulated temperature can yield the temperature resolution of the temperature measurement system.

**Figure 5:**
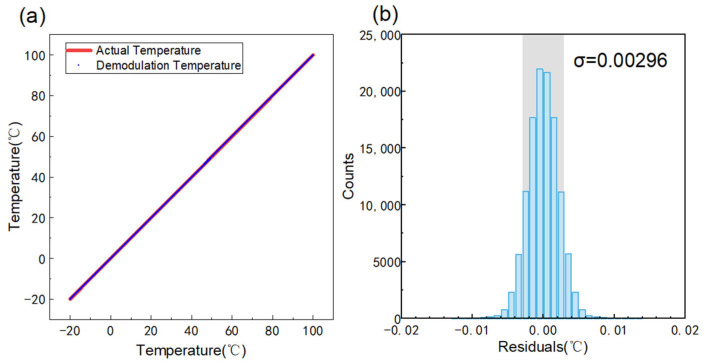
(**a**) Demodulation temperature versus actual temperature. (**b**) Residual distribution of demodulation temperature versus actual temperature, σ = 0.00296 °C.

Figure 5a shows the data of the demodulated temperature versus the actual temperature for the temperature range of −20 °C to 100 °C when the sensor is placed inside the temperature control cabinet.

The residual between the demodulated temperature and the actual temperature is analyzed. Figure 5b demonstrates the residual distribution and the standard deviation σ of the demodulation result is calculated to be 0.00296 °C. However, due to the calibration resolution of 0.001 °C, the final resolution result should be modified to 0.003 °C.

#### 3.3.2. T-Value Uniqueness Analysis

In order to show the uniqueness of the temperature (T-value), we carried out the following data statistics. The four-channel normalized values were used as the four-dimensional coordinates of the points. We calculated the Euclidean distance for all points, and the temperature difference between the nearest points.

**Figure 6:**
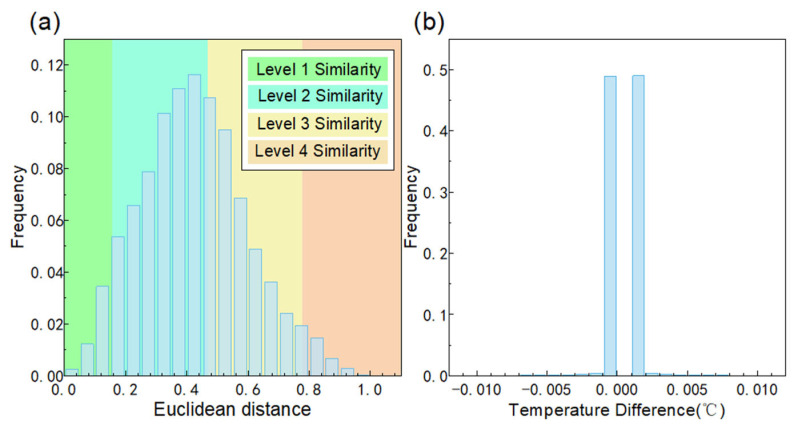
(**a**) Histogram of Euclidean distance. (**b**) Histogram of temperature difference between nearest points.

As shown in Figure 6a, we calculated the distribution of Euclidean distance, where the similarity level was divided according to the following criteria. Euclidean distance 0–0.2 is level 1 similarity, 0.2–0.6 is level 2 similarity, 0.6–1.0 is level 3 similarity, and 1.0 or above is level 4 similarity. The Euclidean distance distribution indicates that within this temperature range, the Euclidean distance exhibits an approximately normal distribution, with 74.29% concentrated in the level 2 similarity.

As shown in Figure 6b, we calculated the nearest points using Euclidean distance. Then we calculated the temperature difference distribution of the nearest points. The histogram of temperature differences shows that 98.32% of the most similar scatters occur within the range of ±0.001°C. This indicates that the majority of the most similar scatters are the closest points in temperature. The above data indicates that the T-value has uniqueness.

#### 3.3.3. Liquid Bath Experiment

**Figure 7:**
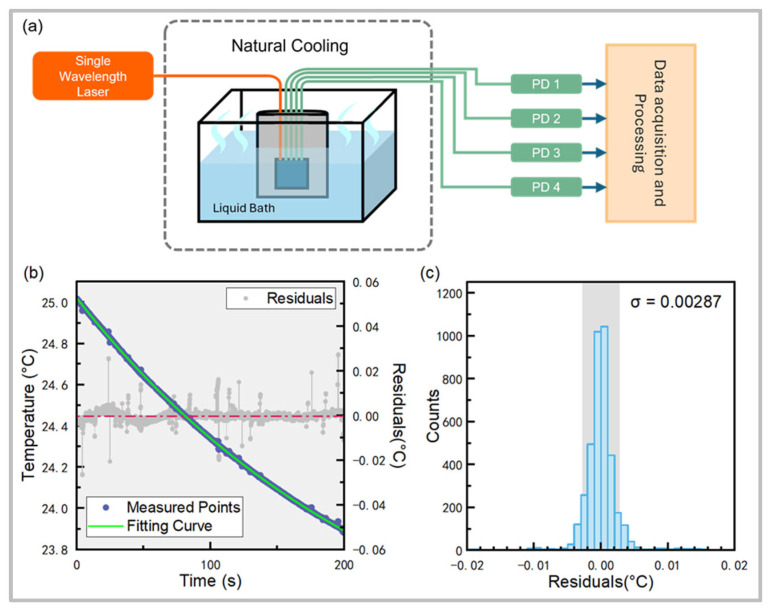
(**a**) Schematic diagram of the device for the natural cooling experiment. (**b**) Measured real-time temperature profile and fitted exponential curve for natural cooling of the probe, fitted R^2^ = 0.9998. (**c**) Residual distribution of measurements versus fitted curve, σ = 0.00287 °C.

As mentioned above, due to the limitation of calibration resolution, it is necessary to demonstrate and calculate a more precise resolution of the sensor by comparing it with the theoretical cooling curve. In addition, the liquid bath experiment is better for demonstrating the reliability of neural network algorithms, since it provides a testing environment that is different from the neural network training environment. Therefore, the measurement resolution of the sensor system was then evaluated by conducting real-time measurements and comparing them with theoretical predictions based on heat transfer principles (as shown in Figure 7a). During testing, the sensor probe which was immerged in a liquid bath was initially heated in a temperature control cabinet and then taken to room temperature for natural cooling. The sensor probe is able to measure the cooling process of the liquid. When the system is stabilized and the ambient temperature is constant, the temperature of the probe declines according to an exponential law [9].

Figure 7a presents the real-time temperature measurements, recorded across 5000 time points over a period of 200 s during the natural cooling process. The observed data align closely with the theoretical exponential cooling curve, with a fitted *R*^2^ value of 0.9998. This high correlation confirms the reliability of the sensor system.

The residuals between the measured data and the theoretical curve were analyzed. Figure 7b shows that the residual distribution and the standard deviation σ of the measurement were calculated to be 0.00287 °C, which indicates that the temperature resolution of the system reaches the 10^−3^ K level.

#### 3.3.4. Aging Performance Experiment

In order to verify the aging performance of the sensor, we conducted a sensor stability test for seven consecutive days with the following experimental procedure. The sensor was placed in the temperature control cabinet, and the temperature control cabinet was set to increase from −20 °C to 100 °C, calculating the residual standard deviation σ according to Figure 5b. And then the process was repeated the next day until the seventh day. The average of the residual standard deviation σ for seven consecutive days was calculated, and the percentage difference between σ and the average for each day was used as the fluctuation value.

**Figure 8:**
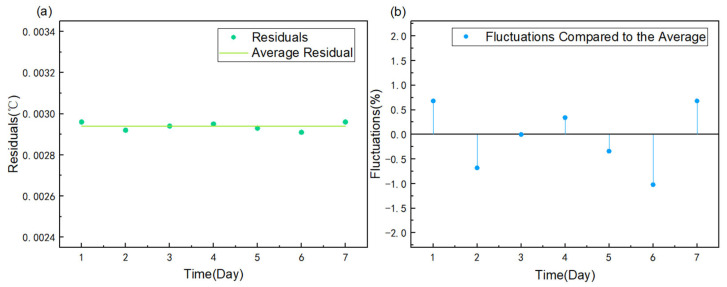
(**a**) Seven consecutive days of stability test data. (**b**) Seven consecutive days of data fluctuations with a maximum fluctuation of 1.02%.

The stability test data are shown in Figure 8a. The average residual is 0.00294 °C within seven days.

The data fluctuation of the seven-day consecutive experiment is shown in Figure 8b. The experiment shows that the maximum value of the fluctuation of the sensor within seven days is 1.02%.

## 4. Conclusions

In this study, we present a highly temperature-sensitive silicon-based integrated temperature sensor system, leveraging the principle of speckle spectroscopy. The system is straightforward and cost-effective. The basic scheme only requires a DFB laser, a silicon optical chip smaller than 1 mm^2^, a five-channel FA for signal transmitting, and four photodetectors. The sensor utilizes a long multimode silicon optical waveguide to enhance temperature sensitivity by exploiting the cumulative effects of the thermo-optic properties of silicon. A taper structure compresses the speckle pattern into four-channel intensity data, simplifying data acquisition and processing. The designed multimode silicon waveguide structure has higher temperature resolution compared to the singlemode silicon waveguide structure. The designed taper structure reduces the cost of acquisition equipment. To achieve precise temperature demodulation, a CNN was employed to address the nonlinear relationship between temperature and light intensity. By training the CNN with a dataset generated during calibration, the system is able to accurately demodulate temperature with a measurement range of about −20 °C to 100 °C and a best temperature resolution of 0.00287 °C (2.87 mK). The system achieves efficient and accurate temperature measurements, with a potential for a wide range of applications. Furthermore, the system’s reliance on a single wavelength for light input suggests the possibility of wavelength-division multiplexing (WDM) to enable distributed sensing. Future work will focus on expanding the system’s capabilities in this direction, exploring its scalability and performance in multiplexed and distributed sensing configurations. In addition, an improved, smooth, bacteria-resistant package may enable the sensor to be an implantable medical device.

## Data Availability

The original contributions presented in this study are included in the article. Further inquiries can be directed to the corresponding author.
